# Pharmacological Modulation of Mutant *TP53* with Oncotargets Against Esophageal Cancer and Therapy Resistance

**DOI:** 10.3390/biomedicines13020450

**Published:** 2025-02-12

**Authors:** Pei-I Lin, Yu-Cheng Lee, I-Hung Chen, Hsien-Hui Chung

**Affiliations:** 1 Department of Nursing, Kaohsiung Chang Gung Memorial Hospital, Kaohsiung City 833401, Taiwan; t3810@cgmh.org.tw; 2 Graduate Institute of Medical Sciences, College of Medicine, Taipei Medical University, Taipei City 110301, Taiwan; yclee0212@tmu.edu.tw; 3 Division of Urology, Department of Surgery, National Cheng Kung University Hospital Douliu Branch, Yunlin County 640003, Taiwan; josephcmed@gmail.com; 4 Department of Pharmacy & Clinical Trial Pharmacy, Kaohsiung Veterans General Hospital, Kaohsiung City 813414, Taiwan; 5 St. Edmund Hall, University of Oxford, Oxford OX1 4AR, UK; 6 Preventive Medicine Program, Center for General Education, Chung Yuan Christian University, Taoyuan City 320314, Taiwan; 7 Department of Pharmacy and Master Program, College of Pharmacy and Health Care, Tajen University, Pingtung County 907101, Taiwan

**Keywords:** esophageal cancer, metabolic modulation, mutant *TP53*, oncotargets, therapy resistance

## Abstract

The prevalence and deaths from esophageal cancer (EC) have recently increased. Although therapeutic strategies depend on the EC stage and recurrence, such as surgical intervention, chemotherapy, radiation therapy, chemoradiation therapy, targeted therapy, and immunotherapy, a more effective and novel treatment for EC is still required. This review briefly describes and summarizes some insightful oncotargets involved in the metabolic modulation of EC, including (1) cancer stem cells (CSCs) for EC progression, poor prognosis, tumor recurrence, and therapy resistance; (2) retinoic acid receptors (RARs) for esophageal carcinogenesis and regeneration; (3) phosphofructokinase (PFK) for EC-reprogrammed glycolysis; (4) lactate dehydrogenase (LDH) as an EC peripheral blood biomarker; and (5) hypoxia-inducible factor-1 alpha (HIF-1α) for the tumor microenvironment under hypoxic conditions. Moreover, the aforementioned oncotargets can be modulated by mutant *TP53* and have their own features in the carcinogenesis, differentiation, proliferation, and metastasis of EC. Thus, the clarification of pharmacological mechanisms regarding the interaction between mutant *TP53* and the abovementioned oncotargets could provide precise and perspective opinions for minimizing prediction errors, reducing therapy resistance, and developing novel drugs against EC.

## 1. Introduction

Esophageal cancer (EC) is a highly prevalent and well-known digestive disease. Recently, EC has become the sixth most common cause of cancer-related deaths worldwide. According to the EC trends from the World Health Organization statistical database, the male age-standardized mortality rate (ASMR)/100,000 in Europe, the United States, and Japan was 4.01, 4.28, and 5.10 in 2015-19, respectively. Additionally, the global range of female ASMRs was from 0.82 to 0.85/100,000 [1].

Patients with EC have difficulty swallowing, accompanied by body weight loss, and risk factors, such as smoking, heavy alcohol use, acid reflux, and Barrett’s esophagus (BE). Esophageal adenocarcinoma (EAC) and esophageal squamous cell carcinoma (ESCC) are the two main types of EC based on histological evaluation and the location of malignancies in the lower and upper esophagus, which are related to transitional and squamous basal cells, respectively. Moreover, the risk factors involved in EAC include obesity, gastroesophageal reflux disease (GERD), single-nucleotide polymorphisms (SNPs), BE, decreased estrogen exposure, and Helicobacter pylori infection. Meanwhile, risk factors involved in ESCC are family history, genetic changes (mutation and SNPs), habits (smoking and drinking), chemical factors, infection, and signal transduction [2]. Clinically, the combination of chemotherapy and radiotherapy can be applied to attenuate EC based on the stages of EC progression. Unfortunately, there is no proper therapeutic strategy once the acquired chemo- or radio-resistance occurs because the failure to treat EC results from chemotherapy resistance linked to complicated mechanisms. Although some promising immunotherapies have attracted much attention, including cancer vaccines for specific targets (preventive type and therapeutic type), immune checkpoint inhibitors (ICIs) for cancer cells and T cells, and chimeric antigen receptor (CAR) T-cell therapy for recognizing specific target antigens, patients with EC receiving immunotherapy have an overall response rate lower than 30% and most patients initially receiving immunotherapy have acquired resistance. Moreover, the immunotherapy resistance in EC still results in clinical challenges owing to drug resistance and immunosuppression associated with oncological characteristics and the heterogeneity of immune microenvironments [3]. Recently, targeting epithelial-mesenchymal conversion by miR-200 has provided a therapeutic direction for ESCC patients with acquired concurrent chemoradiotherapy resistance [4]. Thus, developing therapeutic strategies for improving EC therapy resistance by exploring novel oncotargets and biomarkers is crucial.

## 2. Metabolic Modulation of Oncotargets in Esophageal Cancer

### 2.1. Effects of Cancer Stem Cells on Esophageal Cancer

Generally, normal stem cells represent physiological development, whereas cancer stem cells (CSCs) have distinct morphological features associated with pathological carcinogenesis and tumor-initiating cells. CSCs play crucial roles in the molecular mechanisms of metabolic reprogramming, quiescence, epigenetic modification, progression, poor prognosis, tumor recurrence, and therapeutic resistance in EC. Additionally, impaired mitochondrial function of CSCs, such as imbalanced mitochondrial dynamics, subcellular localization, metabolic plasticity, mitophagy, excessive mitochondria biogenesis, and defective mtDNA, is associated with drug resistance. Moreover, the factors involved in the therapy resistance of esophageal CSCs include DNA repair, the cycle distribution, free radical and reactive oxygen species (ROS) scavenging, the tumor microenvironment, epithelial-mesenchymal transition (EMT), hypoxia, oxidative ROS modulation, autophagy, inflammation, metabolic mechanisms, CSC plasticity, drug transporters, miRNAs, and long noncoding RNAs (lncRNAs) [5]. Some CSC markers—including CD44, CD133, CD271, CD90, leucine-rich repeat-containing G-protein coupled receptor 5 (LgR5), aldehyde dehydrogenase 1 (ALDH1), intercellular cell adhesion molecule-1 (ICAM-1), ATP-binding cassette superfamily G member 2 (ABCG2), integrin subunit alpha 7 (ITGA7), CD44^+^/CD24^−^, CD44^+^/CD133^+^, CD133^+^/CXCR4^+^, B-lymphoma Mo-MLV insertion region 1 (BMI1), NANOG, glioma-associated oncogene homolog 1 (GLI-1), octamer-binding transcription factor 4 (OCT-4), spalt-like transcription factor 4 (SALL4), sex-determining region Y-box2 (SOX2), epithelial cell adhesion molecule (Ep-CAM), and podoplanin—were involved in the diagnosis and prognosis of EC. Moreover, the regulation of CSC proliferation, invasiveness, metastasis, differentiation capacity, and self-renewal in EC may be associated with Notch, Hedgehog, Hippo, Wnt/β-catenin, JAK/STAT3, transforming growth factor-β (TGF-β)/Smad, and phosphatidylinositol 3-kinase (PI3K)/AKT/c-MYC signal transduction pathways [6]. Thus, clarifying metabolic modulation and pathological mechanisms in esophageal CSCs would be helpful for us in finding effective strategies to eradicate EC and control its progression [7]. Most importantly, the clinical targeting CSCs combined with surgical intervention, chemotherapy, radiotherapy, chemoradiation therapy, targeted therapy, and immunotherapy for patients with EC effectively reduce therapy resistance and should be considered.

### 2.2. Effects of Retinoic Acid Receptors (RARs) on Esophageal Carcinogenesis and Regeneration 

Retinoic acid, mainly derived from vitamin A, is involved in physiological cell growth, differentiation, and metabolic homeostasis, which has anti-oxidant, pro-apoptotic, and anti-proliferative effects. Meanwhile, RARs belong to nuclear receptors involved in the modulation of cell functions, including the maintenance of cellular differentiation, proliferation, and survival. Thus, disruption of the retinoic acid pathway by targeting RARs in EC should be elucidated and is worth investigating. Interestingly, in addition to the canonically intracellular role of RARs, a previous study has revealed that the application of exosomes associated with RARs in regenerative medicine, including tissue repair and regeneration, has attracted much attention [8]. Noticeably, activation of RARγ enhances the formation of esophageal epithelial cells (EECs) differentiated from the human-induced pluripotent stem cell (hiPSCs)-derived foregut, providing a novel strategy for regenerative therapy [9]. Nevertheless, the difference in RAR subtypes (RARα, RARβ, and RARγ) involved in esophageal carcinogenesis and regeneration should be further addressed. Thus, I have shared some viewpoints regarding the comparison of RAR subtype-mediated mechanisms in esophageal carcinogenesis and regeneration.

#### 2.2.1. Differential Expressions of RAR Subtypes in Esophageal Carcinogenesis

All-trans retinoic acid (ATRA) has anti-tumor and anti-angiogenesis effects on human ESCC by inhibiting vascular endothelial growth factor (VEGF)-mediated signal transduction [10]. Additionally, CSCs in ESCC that express higher levels of CD44 surface markers are positively correlated with tumorigenicity and chemotherapy resistance. The combination of chemotherapy reagents (cisplatin and 5-fluorouracil) with low concentrations of ATRA enhances tumor cell cycle arrest and apoptosis, exhibiting a synergistic cytotoxic effect against human EC stem cells by suppressing CD44 [11]. As ATRA can non-selectively activate RAR subtypes, the association between distinct RAR subtypes in esophageal carcinogenesis and chemotherapy sensitivity should be investigated. First, it has been demonstrated that RARα was overexpressed in human esophageal carcinoma, whereas silencing RARα expression can attenuate ESCC tumor cell proliferation and metastatic ability through suppressing the Wnt/β-catenin mechanism to resensitize ESCC to chemotherapy treatment [12]. Second, compared to the gain-of-function role of RARα in ESCC tumors, the formation of esophageal carcinogenesis is attributed to the loss of RARβ expressions, but the induction of RARβ expressions in EC cells enhances cell apoptosis via reducing cyclooxygenase-2 expression [13]. Moreover, RARβ silencing in human ESCC associated with hypermethylation of the RARβ promoter region led to the progression and severity of esophageal carcinogenesis, but esophageal carcinogenesis could be ameliorated by DNA methyltransferase inhibitors to partially restore RARβ expressions [14]. 

Furthermore, the other subtype of RARs and RARγ demonstrates no significantly differential expressions in ESCC compared to those in the same matched distant normal tissues [15]. Conversely, patients with smoking and alcohol drinking habits exhibited relatively lower RARγ expressions in ESCC than those in surrounding normal tissues [16], suggesting the possibility of the RARγ response to chronic chemical stimulation and the variation based on different normal tissues. In the above evaluation of the differential expressions of RAR subtype in esophageal carcinogenesis, RARα, RARβ, and RARγ expressions may have similar and distinct characteristics. Moreover, lifestyles, environmental factors, and genetic differences would directly or indirectly alter RAR subtype expressions, which contribute to esophageal carcinogenesis. Using RAR, subtype expressions between patients with EC and healthy humans identified through a genetic analysis of the biobank can be considered as the reference clinical therapy and the identification of compounds applied in pharmacotherapy. The above differential expressions of RAR subtypes in esophageal carcinogenesis are summarized in Table 1.

#### 2.2.2. Differential Expressions of RAR Subtypes During Esophageal Regeneration

Esophageal carcinogenesis is driven by epigenetic mutations, environmental risk factors, GERD, and BE. Although chemotherapy, radiotherapy, and immunotherapy currently serve as the therapeutic options for ESCC treatment, identifying the most effective therapy is essential. Regarding the physiological role of RARs, it is remarkable to mention the beneficial role of ATRA in a single progenitor cell population in the maintenance and repair of the esophageal epithelium, which provides a switching behavior for esophageal regeneration related to wound healing [17].

Surprisingly, the analysis of marker genes during the EEC differentiated process relies on RARγ signaling, not RARα and RARβ, and suitable differentiation protocols. Thus, RARγ activation has a critical and specific impact on esophageal regeneration linked to tissue repair [9]. Furthermore, the application of genetically modified animal models to observe the detailed mechanisms and influence of RARγ linked to the regenerative potential of exosomes on esophageal regeneration is essential because of the complicated interaction of in vivo mimicking that, in humans, has clinical value. Given the above evaluation for the differential expressions of RAR subtype in esophageal regeneration, the stimulation of retinoic acid and RARγ-mediated signaling may induce tissue repair and esophageal regeneration. Thus, retinoic acid synthesis and RARγ expressions involved in esophageal regeneration could provide an alternative therapy against EC and replace abnormal tissues. The differential expressions of RAR subtypes in esophageal regeneration are summarized in Table 1.

#### 2.2.3. Clinical Application of RAR Subtypes in EC

Collectively, the diverse functions and underlying mechanisms of the differential expressions of RAR subtypes in esophageal carcinogenesis or regeneration should be further clarified. The well-understanding in the regulation and mechanisms of distinct RAR subtypes is clinically significant for providing new insights into the combination of anti-carcinogenic action and regenerative therapy through the use of specific RAR subtype agonists or antagonists in patients with ESCC. Thus, the development of RAR subtype compounds in attenuating EC through cellular models, animal models, and clinical trials can be practicably performed. Most importantly, the clinical application of RAR subtype compounds combined with surgical intervention, chemotherapy, radiotherapy, chemoradiation therapy, targeted therapy, and immunotherapy in patients with EC could effectively reduce therapy resistance and is worthwhile to expect.

### 2.3. Metabolic Role of Phosphofructokinase (PFK) in EC

Regarding the utilization of adenosine triphosphate (ATP) for cellular metabolism, compared to normal cells, cancer cells have the preference of glycolysis in the cytosol rather than mitochondrial oxidative phosphorylation based on the requirement of more ATP, which is known as the Warburg effect. When EC exhibits a greater Warburg effect, it is more aggressive and malignant. Thus, focusing on the metabolic reprogramming of EC during glycolysis may help identify potential targets or biomarkers. During the glycolysis process, PFK catalyzes the phosphorylation of fructose-6-phosphate to fructose-1,6-bisphosphate, which is allosterically activated by adenosine monophosphate (AMP) and inhibited by ATP. The aberrant regulation of PFK on glucose metabolism is closely associated with insulin resistance in animal models fed high amounts of fructose [18,19,20]. Currently, two types of PFK have been identified in humans: PFK1 and PFK2. Based on tissue-specific functions and expressions, PFK1 isoforms can be classified as liver-specific PFK (PFKL), skeletal muscle-specific PFK (PFKM), and platelet-specific PFK (PFKP). Although PFK is thought to be associated with cancer progression [21], the metabolic modulation of PFK in EC has not been fully investigated. Patients with ESCC with poor survival and advanced stages had higher PFKL expression levels, but the antipsychotic drug, penfluridol, significantly attenuated ESCC by suppressing PFKL to activate AMP-activated protein kinase (AMPK), forkhead box-containing protein 3a (FOXO3a), and Bcl-2-interacting mediator of cell death (BIM) signaling for glycolysis inhibition and apoptosis induction [22]. In addition, the 6-phosphofructo-2-kinase/fructose-2,6-bisphosphatase isoform 3 (PFKFB3) has biofunctional roles as an effective modulator of glycolytic flux and a critical regulator of angiogenesis, cell death, and cell stemness [23]. An observational study has reported that ESCC tissues exhibited higher protein and gene expression levels of 6-phosphofructo-2-kinase/fructose-2,6-bisphosphatase isoform 3 (PFKFB3) than adjacent non-tumor tissues, indicating the carcinogenic effect of PFKFB3 on the development, occurrence, and prognosis of ESCC. Moreover, the 3-year survival by PFKFB3 mRNA changes could also be predicted in patients with ESCC [24]. However, the genetic absence of PFK or impaired insulin signaling enhanced the extracellular matrix hyaluronan synthesis involved in tumor development and progression by using the nude mouse xenograft model with ESCC induced by streptozotocin, indicating the divergent metabolic regulation of PFK in diabetic EC [25]. Thus, the development of targeting PFK compounds in attenuating EC through cellular models, animal models, and clinical trials can be practicably performed. Certainly, the compounds, by targeting PFK or other related enzymes combined with surgical intervention, chemotherapy, radiotherapy, chemoradiation therapy, targeted therapy, and immunotherapy for patients with EC, could effectively reduce therapy resistance, and this is worthwhile to verify. 

### 2.4. Metabolic Role of Lactate Dehydrogenase (LDH) in EC

The metabolic enzyme LDH is primarily responsible for converting pyruvate to lactate. The enhancement of tumor glycolysis contributes to increased lactate production, which is linked to an acidified tumor microenvironment, metastasis, invasion, angiogenesis, immunosuppression, and therapy resistance. LDH was involved in the tumorigenesis of EC because LDHA was overexpressed in ESCC, which was modulated by decreased cleavage of PARP and caspase 8 and increased AKT activation and cyclin D1 expressions. Relatively, the silencing of LDHA expressions significantly attenuates ESCC migration and growth both in vitro and in vivo [26]. Moreover, some small molecules, such as LDHA antagonists, have been designed for tumor regression and cancer therapeutics, including notinamide adenine dinucleotide and hydrogen (NADH) competitive inhibitors through competition with cofactor NADH; pyruvate competitive inhibitors through competition with substrate pyruvate; and substrate and cofactor competitive inhibitors through competition with substrate pyruvate, as well as NADH, peptides, and galloflavin, which can be potentially considered as EC treatments [27]. Regarding the effects of LDH on therapy resistance, the correlation between LDH levels and EC progression should be investigated. In a single-center retrospective study, patients with ESCC receiving chemoradiotherapy showed that the high-LDH group had shorter overall survival (OS) and progression-free survival (PFS) than the low-LDH group [28]. In a phase 1 clinical trial, compared to patients with normal serum LDH levels at baseline, the increased serum LDH levels at baseline in patients with ESCC exhibited a poorer tumor response, such as complete remission and partial remission, as well as shorter PFS and OS. Moreover, the reduced serum LDH levels are associated with better disease control than the elevated serum LDH levels in patients with ESCC treated with a novel anti-PD-1 antibody, camrelizumab, indicating that the changes of serum LDH levels can be used for evaluating EC prognosis in immunotherapy [29]. Moreover, the metabolic modulation of EC by targeting LDH can be clarified, and the identification of targeting LDH compounds in attenuating EC through cellular models, animal models, and clinical trials can be practicably performed. Furthermore, the compounds, by targeting LDH combined with surgical intervention, chemotherapy, radiation therapy, chemoradiation therapy, targeted therapy, and immunotherapy in patients with EC, can effectively reduce therapy resistance and are worthwhile to evaluate.

### 2.5. Effects of Hypoxia-Inducible Factor-1 (HIF-1) Alpha on EC

HIF-1 is mainly assembled by oxygen-sensitive subunit HIF-1α and constitutively expressed subunit HIF-1β as a heterodimer, and HIF-1α subunit in hypoxia or normoxia can be modulated through post-translation, including phosphorylation, acetylation, hydroxylation, and ubiquitination [30]. The induction of HIF-1 via tumor hypoxia regulates cell survival, angiogenesis, proliferation, invasion, and cancer metabolism. The anaerobic glycolysis is responsible for the metabolic modulation of cancer cells in hypoxic environments. Regarding the effects of HIF-1α expressions on EC, the shorter OS of patients with EC is associated with glycolysis-related proteins and higher HIF-1α expressions [31]. Moreover, the ESCC pathological tumor–node–metastasis stage, T classification, lymph node metastasis, and differentiation can be evaluated via HIF-1α expressions [32]. Compared with local control of low HIF-1α expression (72.5%), poor local control of high HIF-1α expression levels (42.7%) was observed in patients with EC receiving concurrent chemoradiotherapy. The low HIF-1α expression group had a significantly higher 5-year recurrence-free survival (39.8%) than the high HIF-1α expression group (18.2%) [33]. Additionally, the glycolysis of EC in hypoxic conditions may be related to PI3K/AKT and HIF-1α, whereas wortmannin decreased lactic acid and glycolytic enzyme levels (LDHA, hexokinase II, and glucose transporter-1) by inhibiting the PI3K/AKT pathway [34]. The upregulation of G077640, an uncharacterized hypoxia-responsive lncRNA, promotes ESCC proliferation and migration involved in tumorigenesis by interacting with histone H2AX and HIF-1α, which altered hypoxia-related glycolysis, including PDK1, HK2, and GLUT4, but G077640 knockdown attenuates ESCC progression [35]. The C–X–C motif chemokine receptor 4 (CXCR4) antagonist (MSX-122) or CXCR4 knockdown reverses the induction of ESCC migration and invasion by HIF-1α [36]. Furthermore, compared with the absent treatment of berberine, the treatment with berberine significantly increases radiosensitivity in ESCC by blocking VEGF and HIF-1α, which can be clinically applied to radiotherapy resistance [37]. Meanwhile, to observe the tumor microenvironment of EC, non-invasive molecular imaging techniques via ^18^F-HX4 or positron-emission tomography with 2-nitroimidazole derivatives have been used, and some factors for EC, named local control, complete response, disease-free survival, and OS, were evaluated using a hypoxia-associated marker called carbonic anhydrase IX [38]. Thus, the metabolic modulation of EC by targeting HIF-1α can be elucidated, and the identification of targeting HIF-1α compounds in attenuating EC through cellular models, animal models, and clinical trials can be practicably performed. Furthermore, the compounds, by targeting HIF-1α combined with surgical intervention, chemotherapy, radiation therapy, chemoradiation therapy, targeted therapy, and immunotherapy in patients with EC, can effectively reduce therapy resistance and are worth exploring. 

## 3. Metabolic Interaction Between Mutant *TP53* and Oncotargets as a Driver of EC Progression

The mutant *TP53* gene initiates the dysfunction of the tumor suppressor protein p53 in carcinogenesis, metastasis, progression, and therapy resistance in patients with EC [39]. Under the glycolytic metabolism of EAC, missense mutations are the most common type, and the *TP53* mutation frequency is 87% based on the cBioPortal database, greatly affecting the tumor microenvironment and immune response through metabolic reprogramming [40]. p53 modulates cancer metabolism via the liver kinase B1 (LKB1) and AMPK pathways [41], and reduced expression of LKB1 in EAC enhances tumor metastasis and the tumor stage and decreases OS in patients with EAC [42]. However, cross-linking between mutant *TP53* and the aforementioned oncotargets in EC progression has rarely been investigated. Interactions between mutant *TP53* and the aforementioned oncotargets are summarized in Table 2 as follows. First, the *TP53* mutation (R175H mutant p53) enhances proliferation, migration, and invasion in ESCC linked to overexpressed WNT10A and increases the self-renewal capability of CSCs by higher CD44^+^/CD24^−^ population induction [43]. Second, the *TP53* mutation in the patients with EAC downregulates the retinoic acid metabolic process, and adapalene inhibits EAC cell lines through activating RARβ and RARγ [44], indicating the beneficial roles of RARβ and RARγ against esophageal carcinogenesis (Table 1). Third, metabolic reprogramming involved in glycolysis related to ESCC proliferation, invasion, and migration is associated with esophageal *TP53* mutation, accompanied by posttranslational modifications of PFKFB3. Moreover, the enhancement of ESCC proliferation is mediated by the interaction between lncRNA Actin Gamma 1 Pseudogene (*AGPG*) and PFKFB3 against proteasomal degradation [45]. These molecular mechanisms can be applied to develop specific drugs for ESCC patients by targeting the p53-*AGPG*-PFKFB3 axis. Fourth, the overexpression of *TP53*-induced glycolysis and apoptosis regulator (TIGAR) is associated with the progression and therapy resistance of ESCC owing to reduced intracellular lactate levels and metabolic reprogramming from glycolysis to glutamine metabolism [46]. Moreover, the expression of TIGAR and MUC1 transmembrane C-terminal subunit (MUC1-C) in ESCC cells is positively correlated with tumor growth, which is attenuated by targeting MUC1-C and inhibiting the AKT-mTOR-S6K1 pathway [47]. Lastly, the higher HIF-1α expressions under hypoxic regions are positively correlated with higher p53 expressions both in the patients with ESCC and EAC compared to those under normoxic regions [48], which are associated with upregulated TIGAR by HIF-1α in metabolic changes of the tumor microenvironment and therapy sensitivity [49]. Based on the above illustration, pharmacological intervention of mutant *TP53* and its oncotargets may be practical and effective in attenuating EC progression and improving therapy resistance.

## 4. Metabolic Attenuation of Therapy Resistance in EC via Oncotarget Compounds and the Mutant *TP53* Modulator

**Figure 1:**
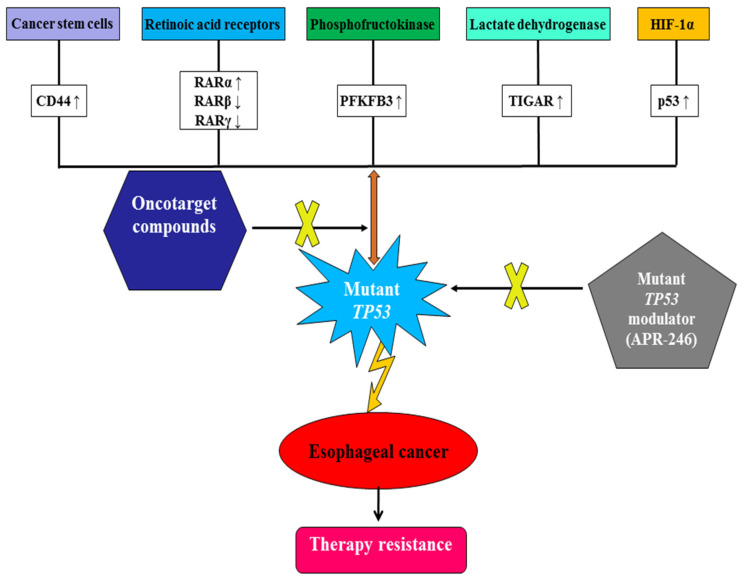
The metabolic attenuation of therapy resistance in esophageal cancer via oncotarget compounds and the mutant *TP53* modulator (APR-246).

Mutant *TP53* can be restored by targeting Cys124 and Cys277 with methylene quinuclidinone converted from APR-246 [50], and eprenetapopt (APR-246) inhibits esophageal cellular proliferation and xenograft tumor growth by inducing ferroptosis, inhibiting NFS1 cysteine desulfurase, and synergizing with dietary serine and glycine restriction [51]. APR-246 has significant antitumor activity and effectively improves chemosensitivity to cisplatin and 5-fluorouracil (5-FU) in preclinical EAC cell lines, cell line xenograft (CLX), and patient-derived xenograft (PDX) models via p53 modification [52]. Moreover, APR-246 induces apoptosis in ESCC cell lines, CLX, and PDX models with a p53 missense mutation through ROS induction and upregulated p73-Noxa signaling, which is enhanced by the combination of 5-FU [53]. In clinical trials, the pharmacotherapeutic sensitivity of APR-246 in patients with EC with the *TP53* mutation was determined by modulating the expressions of solute carrier family 7 member 11 [54]. Thus, the beneficial effects of APR-246 by targeting mutant *TP53* on both preclinical models and clinical trials exhibit therapeutic potential in combating EC and therapy resistance (Table 3). The direct or indirect metabolic regulation and clinical pharmacotherapy in EC by targeting mutant *TP53* and oncotargets should be further verified (Figure 1). 

## 5. Conclusions

Collectively, the multiple integration of CSCs, RARs, PFK, LDH, and HIF-1α with mutant *TP53* provides a promising direction for the precise application of pharmacotherapy, including chemotherapy, gene therapy, chemoradiation therapy, targeted therapy, and immune therapy in the metabolic modulation of therapy resistance due to EC. Most importantly, the algorithmic integration of EC clinical features, biomedical data, genomics, transcriptomics, proteomics, biobank, and clinical trials to develop artificial intelligence models can accelerate the identification of oncotarget compounds and mutant *TP53* modulators as therapeutic drugs to treat EC and improve therapy resistance in the future.

## Figures and Tables

**Table 1:** The comparison of the differential expressions of retinoic acid receptor (RAR) subtype in esophageal regeneration and carcinogenesis.

RAR Subtype	Esophageal Regeneration	Esophageal Carcinogenesis
RARα	Unknown	RARα Overexpression (RARα ↑) [12]
RARβ	Unknown	The loss of RARβ expressions with hypermethylation (RARβ ↓) [14]
RARγ	The differentiation of esophageal epithelial cells from hiPSC-derived foregut through the specific activation of RARγ [9]	Lower RARγ expression (RARγ ↓) [16]

hiPSCs: human-induced pluripotent stem cells.

**Table 2:** The interaction of mutant *TP53* with oncotargets in esophageal cancer.

EC Type	Oncotargets	Main Outcomes	References
ESCC	CSCs	WNT10A was overexpressed in invasive mutant *TP53* cells, and CD44 induction increased self-renewal capability of CSCs	Long et al., 2015 [43]
EAC	RARs	The retinoic acid metabolic process was downregulated in patients with esophageal *TP53* mutation, and the RARβ and RARγ agonist had beneficial effect against EAC cell lines	Zhang et al., 2022 [44]
ESCC	PFK	Esophageal *TP53* mutation induced the activation of glycolysis and the enhancement of cell cycle progression through the upregulation of AGPG and PFKFB3 accumulation	Liu et al., 2020 [45]
ESCC	LDH	The overexpressed TIGAR decreased intracellular lactate levels through the reprogramming of glycolysis to glutamine metabolism for esophageal cancer progression and therapy resistance	Chu et al., 2020 [46]
ESCC and EAC	HIF-1α	HIF-1α upregulated p53 expressions in hypoxic regions compared to normoxic regions	Igbo et al., 2022 [48]

AGPG: Actin Gamma 1 Pseudogene; PFKFB3: 6-phosphofructo-2-kinase/fructose-2,6-bisphosphatase isoform 3; TIGAR: TP53-induced glycolysis and apoptosis regulator.

**Table 3:** The amelioration of esophageal cancer by restoring *TP53* mutation with APR-246.

EC Type Models	Main Findings	References
EAC cell lines and Xenograft mice models	APR-246 inhibited esophageal cancer by inducing ferroptosis, inhibiting NFS1 cysteine desulfurase, and synergizing with dietary serine and glycine restriction	Fujihara et al., 2022 [51]
EAC cell lines, CLX and PDX models	APR-246 inhibited EAC cell survival, induced apoptosis and enhanced chemosensitivity to a cisplatin/5-FU-resistant xenograft model	Liu et al., 2015 [52]
ESCC cell lines, CLX and PDX models	APR-246 induced apoptosis in ESCC with p53 mutation through ROS induction and p73-Noxa signaling, which was enhanced by the combination of 5-FU	Kobayashi et al., 2021 [53]
EC patients	The sensitivity of APR-246 to EC with *TP53* mutation can be determined by the modulation of SLC7A11 expressions	Fujihara et al., 2021 [54]

5-FU: 5-fluorouracil; CLX: cell line xenograft; PDX: patient-derived xenograft; ROS: reactive oxygen species; SLC7A11: solute carrier family 7 member 11.

## Data Availability

No new data were analyzed or created in this study, and data sharing was not applicable to this article.
